# The metabolic cost of nesting: body condition and blood parameters of *Caiman crocodilus* and *Melanosuchus niger* in Central Amazonia

**DOI:** 10.1007/s00360-017-1103-8

**Published:** 2017-06-19

**Authors:** José António Lemos Barão-Nóbrega, Boris Marioni, Robinson Botero-Arias, António José Arsénia Nogueira, Emerson Silva Lima, William Ernest Magnusson, Ronis Da Silveira, Jaydione Luiz Marcon

**Affiliations:** 1 Departamento de Biologia, Universidade de Aveiro - UA, Campus de Santiago, 3810-193 Aveiro, Portugal; 2 Programa de Pós-Graduação em Ecologia – Instituto Nacional de Pesquisas da Amazónia (INPA), CP 478, Manaus, AM 69011-970 Brazil; 3 Programa de Conservação de Crocodilianos Amazónicos – Instituto Piagaçu, Rua UZ no. 8 Cj Morada do Sol, Manaus, AM 6900-000 Brazil; 4 Programa de Pesquisa em Conservação e Manejo de Jacarés – Instituto de Desenvolvimento Sustentável Mamirauá, Estrada do Bexiga, 2584 - Bairro Fonte Boa, Tefé, AM CEP 69470-000 Brazil; 5 Department of Wildlife Ecology and Conservation, Institute of Food and Agricultural Sciences, University of Florida, 110 Newins-Ziegler Hall, PO Box 110430, Gainesville, FL 32611-0430 USA; 6 Centro de Estudos do Ambiente e do Mar - CESAM - UA, Campus de Santiago, 3810-193 Aveiro, Portugal; 7 Laboratório de Atividade Biológica, Faculdade de Ciências Farmacêuticas, Universidade Federal do Amazonas, Av. Gen, Rodrigo Otávio 6200, Manaus, AM CEP 69077-070 Brazil; 8 Coordenação de Biodiversidade, Instituto Nacional de Pesquisas da Amazónia (INPA), CP 2228, Manaus, AM 69080-971 Brazil; 9 Laboratório de Zoologia-Aplicada à Conservação, Departamento de Biologia, Instituto de Ciências Biológicas, Universidade Federal do Amazonas, Av. Gen, Rodrigo Otávio 6200, Manaus, AM CEP 69077-070 Brazil; 10 Laboratório de Fisiologia Animal, Departamento de Ciências Fisiológicas, Instituto de Ciências Biológicas, Universidade Federal do Amazonas, Av. Gen, Rodrigo Otávio 6200, Manaus, AM CEP 69077-070 Brazil

**Keywords:** Amazon, Blood, Body condition, Caiman, Nest, Várzea

## Abstract

Although nesting ecology is well studied in several crocodilian species, it is not known how nest attendance influences physiology and body condition of nesting females. In this study, we describe body condition and serum biochemical values of nesting female, non-nesting female and male spectacled caiman (*Caiman crocodilus*) and black caiman (*Melanosuchus niger*) in two areas of Central Amazonia. We also evaluated the effect of nest age and nest distance to water on body condition and blood parameters of nesting females. Body condition and plasmatic concentrations of glucose, triglycerides, lactate and uric acid of nesting females were significantly different from those of non-nesting females and males in *C. crocodilus*, but not in *M. niger*. Our study also demonstrated that nest age and distance to water had a negative effect on female body condition in *C. crocodilus*, but not in *M. niger*. Female *C. crocodilus* attending older nests or nests built further away from permanent water bodies tended to have lower body condition. Our results demonstrate that the nesting strategy of *C. crocodilus* has a metabolic cost associated with nest attendance for nesting females, which appear to depend on accumulated energetic reserves during nest attendance. In contrast, nest attendance had little effect on the physiology of female *M. niger*.

## Introduction

Body condition analysis is a non-destructive and non-invasive method that has proven valuable in ecological fields of many invertebrate (Moya-Laraño et al. 2008) and vertebrate species (Kitaysky et al. 1999; Stevenson and Woods 2006; Mazzotti et al. 2009). Body condition of crocodilians (Zweig et al. 2014) has been used in studies of the effects of environmental variables (Fujisaki et al. 2009; Mazzotti et al. 2012), diet (Santos et al. 1996; Delany et al. 1999), growth (Saafeld et al. 2008) and haematological and parasitological parameters (Padilla-Paz 2008). However, there is no available information on how nest attendance may influence physiology and body condition of nesting female crocodilians.

At least three of the four crocodilian species that occur in the Amazon basin construct their mound nests in várzea (seasonal flooded forest) habitats during the annual dry season, when the water levels are at their lowest values (Thorbjarnarson et al. 2000; Villamarín et al. 2011). When gravid females migrate to nesting areas, males and non-nesting females remain in the main water canals (Thorbjarnarson 1994; Ayarzagϋena and Castroviejo 2008). The spectacled caiman (*Caiman crocodilus*) is the species with the most generalist nesting strategy; some nests are built along the margins of lakes and canals, while others are deep inside the forest, sometimes at distances of hundreds of metres from a permanent water body (Da Silveira et al. 2010; Villamarín et al. 2011). Nesting female *C. crocodilus* usually remain hidden in vegetation near the nest, frequently under leaf litter or in the debris of fallen trees, where they attend the nest in an energy-conserving (they endure periods of food deprivation while attending nests; Barão-Nóbrega et al. 2016) lethargic state during the entire incubation period (Staton and Dixon 1977; Marioni et al. 2007). In contrast, nesting females of the sympatric black caiman (*Melanosuchus niger*) construct their nests close to the water, usually near lakes isolated from the main water canal during the dry season (Da Silveira et al. 1997; Villamarín et al. 2011). Female *M. niger* remain near their nests during the egg incubation period, usually in the water (where they have access to food resources), and often defend them aggressively (more energy demanding than just attending) against potential predators (Thorbjarnarson et al. 2000).

Body and physiological condition of nesting females (and thus nest attendance behaviour) are likely to depend on how long they have been attending the nest and how difficult it is for them to reach water, which depends on how far the nest is into the forest. As nesting *C. crocodilus* often uses terrestrial habitats far from water, it is important to consider the influence of dehydration on body condition analysis (Lane 1996; Coppo et al. 2006). The American alligator (*Alligator mississippiensis*) loses water due to evaporation at a rate that is directly related to temperature and inversely related to body size (Ross 1989). Without access to water, young crocodilians may lose up to 20% of their body weight per day (Coppo et al. 2006).

Although the effects of nest attendance on the nutritional and physiological condition of nesting female caimans have not been assessed previously, they can be evaluated by measuring the plasma concentrations of certain metabolites, such as glucose, lactate, and plasmatic triglycerides, which are the main source of metabolic energy in crocodilians (Black et al. 1963). For example, *C. yacare*, which endures periods of food deprivation due to not having immediate access to water in the dry season, exhibited significant reduction in plasmatic glucose and triglycerides (Campbell et al. 2008). Thus, measuring these metabolites provides a better understanding of the mobilization of energy by nesting females during nest attendance and to what extent are they relying on their accumulated reserves (Robin et al. 1988; Campbell et al. 2008). Similarly, attending nests further from water may result in higher dehydration rates in nesting females, especially in *C. crocodilus*. Dehydration can be evaluated by measuring plasmatic concentrations of total proteins (which if in low concentration provide insight into whether protein catabolization is being used as an additional energy source; Campbell et al. 2008) and uric acid, which is the main means of nitrogen excretion in dehydrated crocodilians (Khalil and Haggag 1958; Coulson and Hernandez 1959).

The female’s physiological state while attending the nest may influence her response to stress factors, such as nest predation attempts or capture, immobilization and post-capture handling procedures (Lance et al. 2000; Franklin et al. 2003). Therefore, monitoring and conservation programs that involve nest surveys and/or capture of attending females (e.g. Campos et al. 2008) should take into account the probable physiological condition of the female.

This study reports on the influence of nest attendance on body condition and physiological state of female caimans, and how these are affected by nesting strategy, by comparing two species (*C. crocodilus* and *M. niger*) in Central Amazonia. We hypothesized that (1) nesting females would exhibit lower body condition than non-nesting individuals in *C. crocodilus*, but not in *M. niger*; (2) caimans attending nests would have different serum biochemical values than those not attending nests in *C. crocodilus*, but not in *M. niger*; (3) body condition would reflect the results observed in the blood biochemistry analysis; (4) nest distance from standing water or time in attendance at the nest would have a negative effect on body condition and serum biochemical values of nesting females.

## Materials and methods

### Study areas

**Fig. 1:**
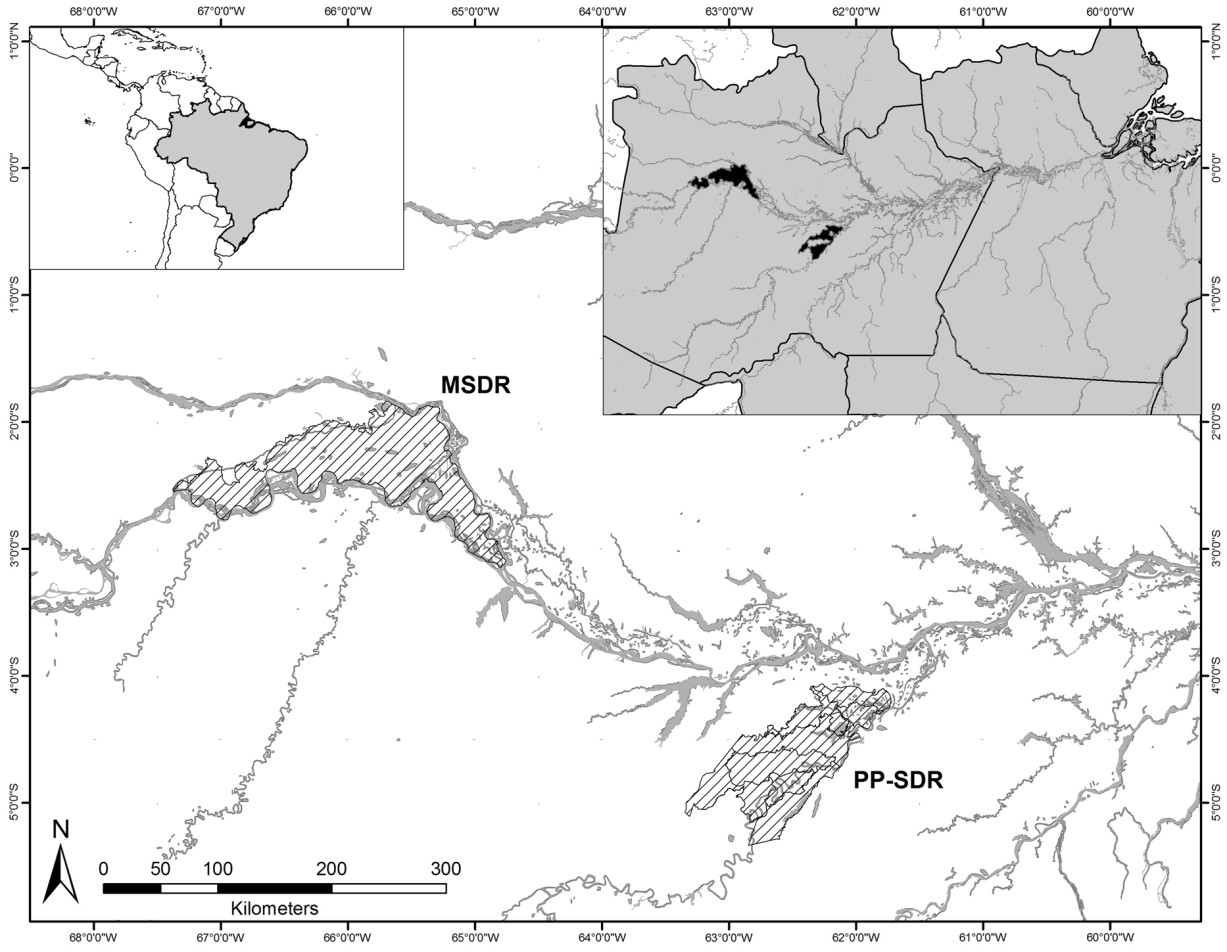
Location of Piagaçu-Purus sustainable development reserve (PP-SDR, our main site) and Mamirauá (MSDR) sustainable development reserve, Central Amazonia

Our study was conducted in two sites in Central Amazonia (Fig. 1). These areas enclose várzea floodplains and are highly dynamic hydrological systems influenced by annual flooding (Wittmann et al. 2004; Junk et al. 2010). The main part of our study was carried out in the Piagaçu-Purus sustainable development reserve (PP-SDR), which is located between the Purus and Amazonas Rivers, approximately 350 km southwest of Manaus, Amazonas State, Brazil. PP-SDR encompasses about 834,245 ha. Várzea covers about half of the reserve, and includes lakes and canals covered by floating vegetation, as well as forest (Haugaasen and Peres 2006). The second part of the study was conducted in Mamirauá sustainable development reserve (MSDR), located between the Solimões (Amazon) and Japurá Rivers, approximately 250 km northwest of PP-SDR. MSDR encompasses an area of 1,124,000 ha, and is entirely composed of várzea floodplains (Mamirauá 1995).

Although species-specific nesting strategies are the same in both study areas (Villamarín et al. 2011), there is a predominance of *C. crocodilus* nests in PP-SDR, and *M. niger* in MSDR. The heterogeneous spatial distribution of these two crocodilians has been suggested to be the result of past hunting pressure (e.g. Magnusson 1985). Nonetheless, it is unlikely that the nesting strategy of *C. crocodilus* and *M. niger* (the focus of this study) in either area is shaped by past hunting pressure (Villamarín et al. 2011). In Central Amazonia, *M. niger* and *C. crocodilus* also show spatial segregation, ecological differences in habitat selection and resource use (Da Silveira et al. 1997; Magnusson 1985; Marioni et al. 2008).

### Searching for nests

Nest surveys were carried out in 2012 from October to November in MSDR and between November and December in PP-SDR. In both sites, this period coincides with the annual dry season, when water levels are at their lowest and caimans are nesting. For both species, nesting starts in late September with the latest nests being built around mid-November (Villamarín et al. 2011). In PP-SDR, nests were located by walking the margins of water bodies and their adjacent areas of várzea forest, up to a distance of 100 m into the forest (Marioni et al. 2007). In MSDR, nest surveys were carried out up to 20 m landward of the water’s edge, which is where most *M. niger* nests are located (Villamarín et al. 2011).

We surveyed 14 water bodies in PP-SDR and found 118 nests of *C. crocodilus* and 4 nests of *M. niger*. We were only able to locate the attending females in 49 nests of *C. crocodilus*. In MSDR, we located 45 *M. niger* and 6 *C. crocodilus* nests in 8 water bodies, but were only able to locate 15 attending females of *M. niger* and 3 females of *C. crocodilus*. Because of the low sample size for *C. crocodilus* nesting females in MSDR, we did not include them in the analysis.

Straight-line distance from the nest to the forest edge in the direction of the nearest water body was measured with a hip-chain^®^ (Forestry Suppliers, Inc, Jackson, Mississippi, USA) for each nest. Distance to water beyond the forest edge was not used to indicate distance from water as this varied during incubation. Coordinates of nests were registered with a Garmin^®^ 78 S GPS recorder.

One egg from each *C. crocodilus* nest was opened on the same day the respective female was captured and the curled length (Crawshaw 1987; Campos et al. 2008) of the embryo measured with Vernier callipers (±0.05 mm). Embryo length was used as an index of nest age in analyses (Campos et al. 2008). As there is no information available on the relationship between embryo length and incubation time for *C. crocodilus* in Central Amazonia, our estimate of nest age was based on the relationship described for *Caiman yacare* in the Brazilian Pantanal, (Crawshaw 1987). Nest age of *M. niger* was not estimated, as embryo length could not be measured.

### Female capture

Nesting females of both species were captured near the nest and physically restrained using a pole-snare (Ketch-All Animal Restraining Pole), ropes and tapes. We measured snout–vent length (SVL) and body mass, and marked the caiman by removal of a combination of three vertical tail scutes (Da Silveira et al. 1997). Females not attending nests (non-nesting) were located by their eyeshine when illuminated with a spotlight during nocturnal surveys, conducted from an aluminium boat with a 15 HP motor in canals with permanent water flow, locally called paranãs (Ayarzagüena 1983; Thorbjarnarson 1994). In PP-SDR, most females with SVL >60 cm are mature (Souza et al. 2010). Adult males of similar size (60 ≤ SVL ≤ 90) were also captured to provide an additional comparison with nesting and non-nesting females of *C. crocodilus*. In MSDR, all individuals of *M. niger* encountered in the canals were captured. Males and non-nesting females of both species were captured using a pole with a break-away noose, immobilized, measured, weighed and marked as described for nesting females (Da Silveira et al. 1997). Sex was identified by direct examination of the cloaca (Ziegler and Olbort 2007). All captured caimans were released within a maximum period of 30 min after capture.

### Blood samples

Blood samples were collected within a maximum period of 15 min after capture (Guillette et al. 1997), immediately after physical immobilization and before the measuring and weighting procedures described above. Approximately 5 mL of blood was withdrawn from the supravertebral sinus (Sykes and Klaphake 2008) into a sterile syringe using a 22G × 1″ needle (0.7 × 25 mm). For larger individuals of *M. niger*, a 21G × 1″ needle (0.8 × 38 mm) was used. After collection, blood was transferred to tubes containing lithium heparin (BD Vacutainer™, São Paulo, Brazil) and kept in ice until processed.

All blood samples were processed immediately upon our arrival at our base camps in PP-SDR and MSDR. Initial processing was performed in improvised laboratories at both sites. Blood was centrifuged at 6400 rpm for five min. Plasma was transferred to a cryogenic tube (2 mL) and immediately frozen in liquid nitrogen. The time between blood collection and plasma separation ranged from one to twelve hours (mean ± SD = 5 ± 3 h). Plasma samples were then transported to Manaus and stored at −85 °C in an ultrafreezer (Sanyo Corp., New York, USA) until further analysis.

### Body condition analysis

To determine whether Fulton’s *K* body condition index, which assumes isometric growth (Green 2001), could be used for this study, we calculated the regression slope (*b*) of the natural log of length on the natural log of mass with our data and tested whether it was significantly different from 3 (Zar 1999). In *C. crocodilus*, the regression slope (*b*) for all 92 caimans captured during our study was significantly different from three (*b* = 2.950; *p* < 0.05). However, as the regression slope (*b*) is very close to three, and the slope (*b*) of the 43 caimans not affected by the costs associated with reproduction alone (non-nesting females and adult males) was not significantly different from 3 (*b* = 3.003; *p* = 0.17), we used Fulton’s *K* as the body condition index for all individuals of this species. When the slope (*b*) value is close to three, the calculations for Fulton’s *K* and relative condition *K*_*b*_ (LeCren 1951) are almost equal (Mazzotti et al. 2012). Furthermore, it was already demonstrated for *Alligator mississippiensis* that Fulton’s *K* is capable of showing differences in body condition either using the isometric growth value (*b* = 3) or the real value (*K*_*b*_), even when slope (*b*) is significantly different from 3 (Zweig 2003). Thereby, caiman body condition in *C. crocodilus* was estimated according to the following equation: *K* = (body mass/SVL^*b*^) × 10^*n*^, where *b* = 3 and *n* = 5 (Green 2001; Nash et al. 2006; Zweig et al. 2014). As the slope (*b*) was significantly different from three (*b* = 2.93; *p* < 0.05) in *M. niger*, and exhibited the same value in both situations (with and without nesting females), body condition was evaluated by the relative condition index (*K*_*b*_), according to the following equation: *K*_*b*_ = (body mass/SVL^2.93^) × 10^5^ (LeCren 1951).

### Biochemical analysis

Biochemical analysis was performed in a Cobas Mira Plus automated system (Roche^®^, Basel, Switzerland). Plasmatic glucose, triglycerides, lactate, uric acid and protein (total) concentrations were determined using specific commercial kits of colorimetric reaction (Glicose liquiform, Triglicérides liquiform, Lactato liquiform, Ácido Úrico liquiform and Proteínas Totais). All reagents were purchased from the same supplier (LabTest™, Lagoa Santa, Minas Gerais, Brazil) and procedures were performed according to the manufacturer’s instructions. All metabolites were measured in duplicate. Lactate concentration could only be measured in *C. crocodilus*.

### Statistical analysis

All statistical analysis and graphics was performed in Systat 8.0 statistical software (Systat 8.0, SPSS Inc., Chicago). Analysis of variance (ANOVA) followed by a Tukey’s post hoc test (*p* < 0.05) was used to evaluate whether there were significant differences in body condition between nesting and non-nesting individuals of *C. crocodilus*. In *M. niger*, SVL was added as a covariate in the analysis (ANCOVA) to account for differences in size between nesting and non-nesting individuals (*F* = 21.39; *p* < 0.001). Differences in blood biochemistry parameters between nesting females, non-nesting females and males were assessed by multivariate analysis of covariance (MANCOVA). Plasmatic concentrations of glucose, triglycerides, lactate, uric acid and total proteins were square-root transformed to meet the normality assumption of this test (Zar 1999). As there were significant differences in blood collection time (time between the beginning of capture effort and blood withdrawal) between captures near nests and far from nests for *C. crocodilus* (Mann–Whitney *U* = 87.0; *p* < 0.001), time to blood removal was included in the analysis as a covariate for that species. In *M. niger*, there was no significant difference in blood collection time between nesting and non-nesting individuals (Mann–Whitney *U* = 127.0; *p* = 0.200), due to the small sample size in all four analysed blood parameters (glucose and triglycerides: *n* < 10; total proteins and uric acid: *n* < 5) in non-nesting black caimans, we included males and non-nesting females in a single group, and total proteins and uric acid were analysed separately from glucose and triglycerides.

Multiple regression analysis was used to examine if body condition influenced serum biochemical values, with nesting and non-nesting individuals being analysed separately, and to examine the influence of nest variables (age and distance) on nesting female body condition and blood parameters. This multivariate analysis was followed by linear regression when we suspected the effect on a single blood parameter. Uric acid and total protein concentrations were not included in the analysis for non-nesting female and male *M. niger* because the number of samples was less than five.

## Results

### Nest surveys

Nest distance to forest edge ranged from 14 to 100 m (mean ± s.d. = 55 ± 31 m) in *C. crocodilus* and from zero to 8 (mean ± s.d. = 1.3 ± 2 m) in *M. niger*. Curled embryo length ranged from 46.1 to 239.0 mm (mean ± s.d. = 163.7 ± 51.5 mm) for *C. crocodilus*, which corresponds to a nest age variation between 12 and 65 days (mean ± s.d. = 44 ± 14 days).

### Body condition analysis

**Fig. 2:**
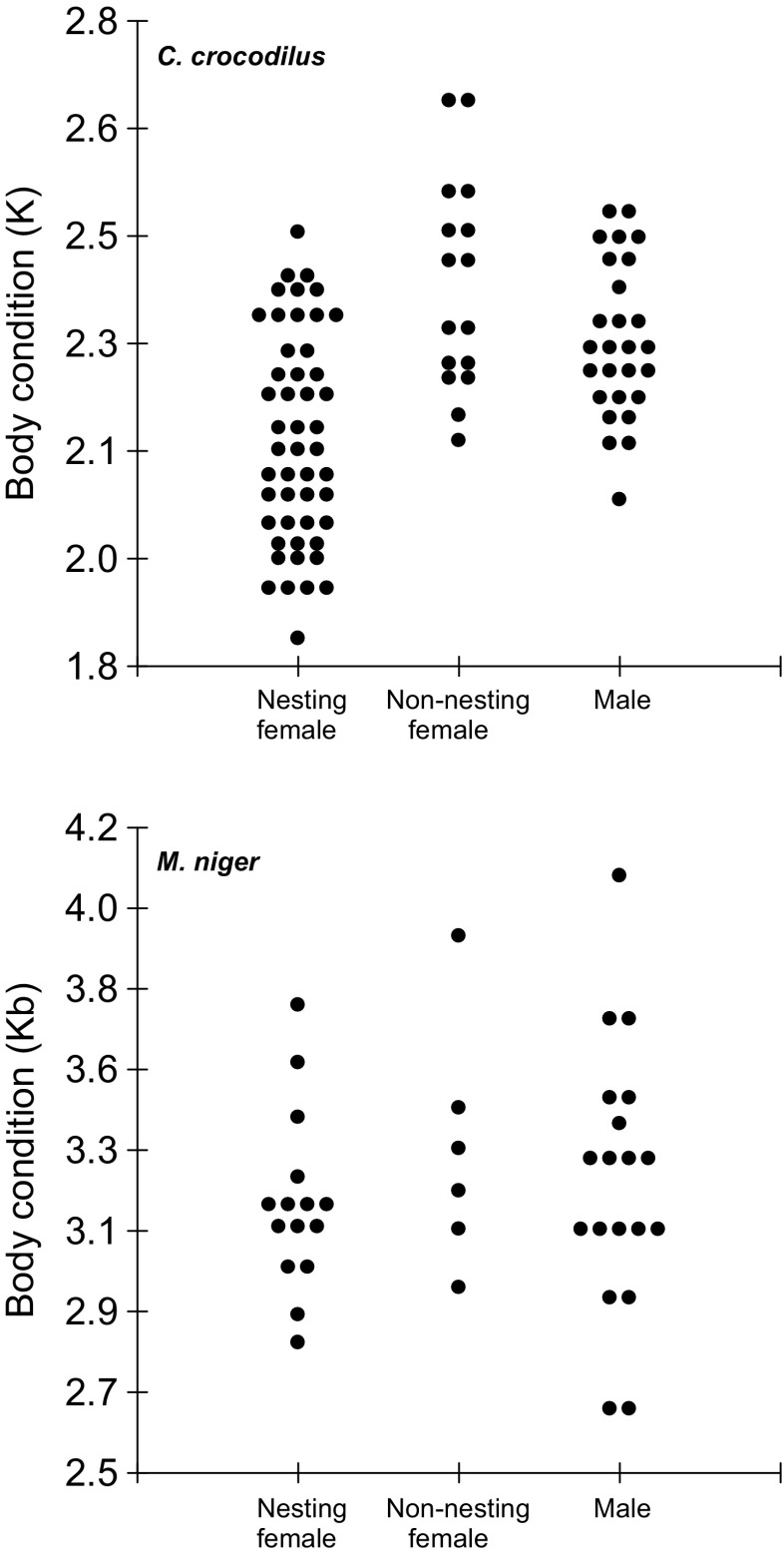
Body condition of females captured near the nest (nesting females), females captured away from nests (non-nesting females) and males of *Caiman crocodilus* (Fulton’s *K*) in PP-SDR and *Melanosuchus niger* (*K*_*b*_) in MSDR. Each point represents a caiman

There was no significant differences in SVL between nesting and non-nesting *C. crocodilus* (*F* = 2.84; *p* = 0.070; Table 1). Fulton’s “*K*” body condition of nesting females was significantly lower than non-nesting females and adult males (*F* = 18.63; *p* < 0.001). However, no significant difference was observed in body condition between non-nesting females and adult males (*p* = 0.150; Fig. 2a). In *M. niger*, no significant difference (*F* = 0.57; *p* = 0.569) was observed in *K*_*b*_ body condition between nesting and non-nesting individuals of *M. niger* (Fig. 2b), and there was also no effect of SVL (*F* = 0.19; *p* = 0.665).

**Table 1:** Morphological measurements, body condition scores and biochemical data for nesting females, non-nesting females and males of *Caiman crocodilus* in PP-SDR and *Melanosuchus niger* in MSDR, Central Amazonia

Parameter	*Caiman crocodilus*						*Melanosuchus niger*					
	Nesting female		Non-nesting female		Male		Nesting female		Non-nesting female		Male	
	*N*	Mean ± s.d (min–max)	*N*	Mean ± s.d (min–max)	*N*	Mean ± s.d. (min–max)	*N*	Mean ± s.d (min–max)	*N*	Mean ± s.d. (min–max)	*N*	Mean ± s.d (min–max)
SVL (cm)^b^	49	76.5 ± 5.0 (65.8–92.0)	16	72.3 ± 6.3 (61.0–79.8)	27	76.3 ± 8.0 (62.4–92.4)	15	135 ± 7 (124–147)	6	88 ± 20 (58–108)	19	81 ± 31 (49–138)
Mass (kg)^b^	49	9.7 ± 2.0 (5.9–16.6)	16	9.3 ± 2.4 (5.1–13.4)	27	10.5 ± 3.2 (5.0–17.4)	15	56 ± 9 (43 ± 79)	6	19 ± 12 (5–36)	19	18 ± 20 (3.2–65)
Body condition^a^	49	2.2 ± 0.2 (1.8–2.5)	16	2.4 ± 0.2 (2.2–2.7)	27	2.3 ± 0.1 (2.1–2.5)	15	3.2 ± 0.2 (2.8–3.7)	6	3.4 ± 0.3 (3.0–3.9)	19	3.2 ± 0.3 (2.7–4.1)
Glucose^a^ (mmol/L)	43	2.8 ± 0.7 (1.3–4.6)	16	4.9 ± 1.9 (2.8–10.4)	14	5.1 ± 1.0 (3.4–7.0)	15	4.4 ± 1.2 (2.7–7.1)	6	4.7 ± 0.1 (3.3–5.8)	9	5.1 ± 0.8 (4.1–6.3)
Protein total (g/L)	43	76 ± 19 (22–111)	16	74 ± 15 (47–98)	14	70 ± 14 (35–86)	7	63 ± 48 (54–67)	2	64 ± 6 (60–68)	3	48 ± 6 (41–54)
Triglycerides^a^ (mmol/L)	43	0.4 ± 0.1 (0.1–0.8)	16	1.6 ± 0.9 (0.3–3.7)	14	1.7 ± 1.3 (0.2–4.3)	15	0.6 ± 0.4 (0.2–1.7)	6	0.7 ± 0.4 (0.4–1.5)	9	0.4 ± 0.2 (0.05–0.8)
Uric acid^a^ (µmol/L)	43	340 ± 162 (36–797)	16	151 ± 62 (59–280)	14	166 ± 91 (77–375)	7	101 ± 38 (54–172)	2	89 ± 34 (65–113)	3	99 ± 19 (77–113)
Lactate^a^ (mmol/L)	43	2.9 ± 1.5 (0.6–6.7)	16	6.0 ± 1.6 (2.5–8.9)	14	5.4 ± 1.8 (1.7–8.1)	–	–	–	–	–	–

Numbers of observations (*N*) may vary because not all parameters could be evaluated for every caiman. “a” and “b” represent significant difference (*α* < 0.05) between nesting and non-nesting individuals in *C. crocodilus* and *M. niger*, respectively. Body condition in *C. crocodilus* and *M. niger* was estimated, respectively, through Fulton’s “*K*” and relative condition *K*_*b*_ (see M&M for details), and so presented values cannot be compared between species

### Biochemistry analysis

**Fig. 3:**
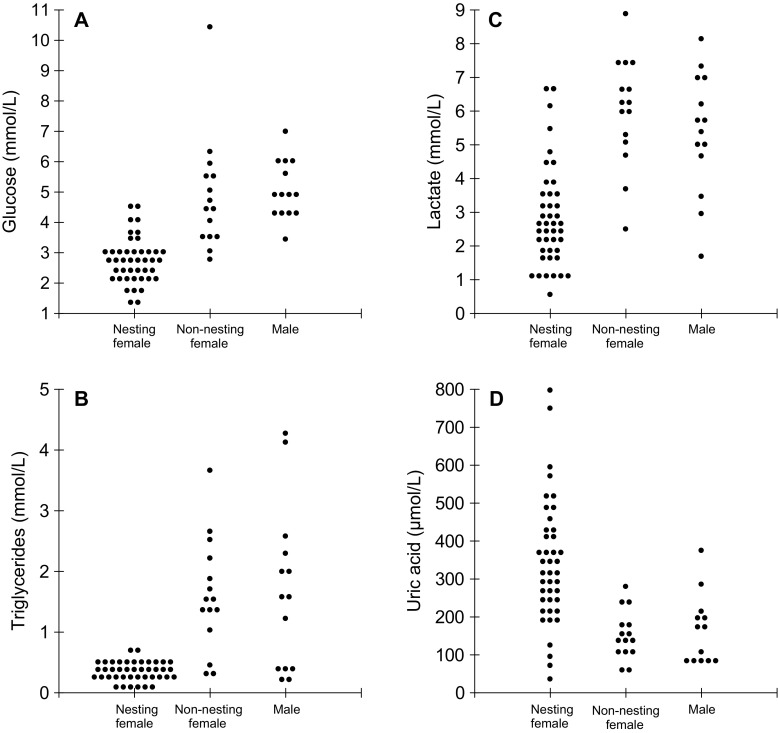
Plasmatic concentrations of glucose (**a**), triglycerides (**b**), lactate (**c**) and uric acid (**d**) of females captured near the nest (nesting females), females captured away from nests (non-nesting females) and adult males of *Caiman crocodilus* in PP-SDR. Each point represents a caiman. In all four blood parameters, significant difference (*p* < 0.05) was observed between nesting and non-nesting caimans, but not between non-nesting females and males

Descriptive information from the blood plasma biochemistry analyses for *C. crocodilus* and *M. niger* are listed in Table 1. In *C. crocodilus*, MANCOVA indicated that animal group (nesting female, non-nesting female or adult male) affected four of five blood plasma biochemistry parameters (*F* = 11.84; *p* < 0.001; Fig. 3), but there was no effect of blood plasma collection time in any group (*F* = 1.07; *p* = 0.384). Plasmatic concentrations of glucose (*F* = 27.28; *p* < 0.001), triglycerides (*F* = 32.78; *p* < 0.001), lactate (*F* = 18.18; *p* < 0.001) and uric acid (*F* = 11.26; *p* < 0.001) differed between nesting and non-nesting individuals, but there was no significant difference between non-nesting females and adult males (glucose: *p* = 0.720; triglycerides: *p* = 0.970; lactate: *p* = 0.840; uric acid: *p* = 0.970). No significant difference was observed for plasmatic total protein concentration (*F* = 0.69; *p* = 0.506) between nesting and non-nesting individuals of *C. crocodilus*.

**Fig. 4:**
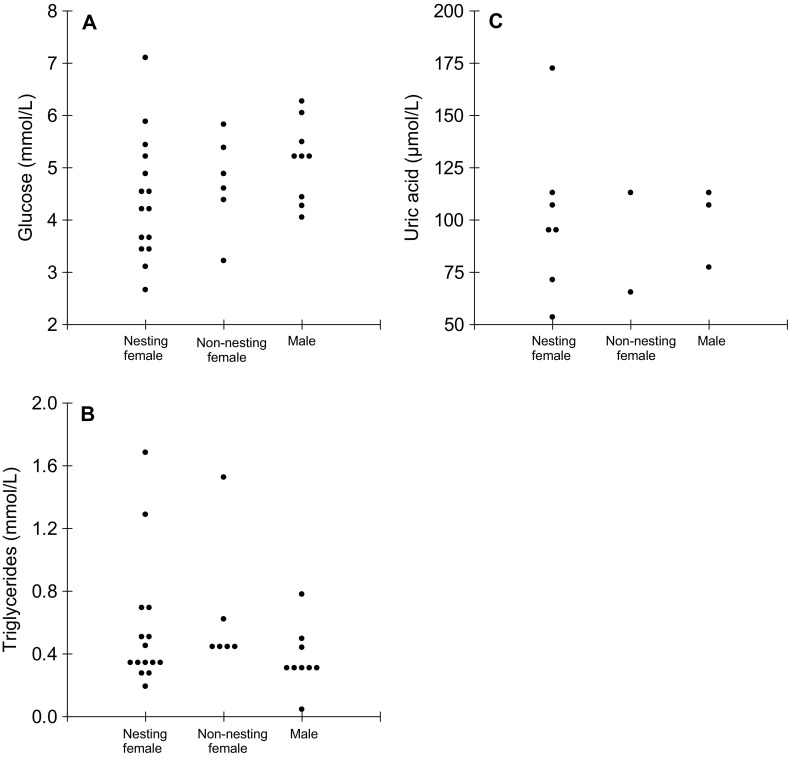
Plasmatic concentrations of glucose (**a**), triglycerides (**b**) and uric acid (**c**) of females captured near the nest (nesting females), females captured away from nests (non-nesting females) and males of *Melanosuchus niger* in MSDR. Each point represents a caiman

For *M. niger*, MANCOVA indicated no significant difference between nesting and non-nesting caimans (*F* = 1.07; *p* = 0.356; Fig. 4) in plasmatic concentrations of glucose (*F* = 1.18; *p* = 0.288) and triglycerides (*F* = 0.70; *p* = 0.411), but plasmatic concentration of glucose had a significant relationship with SVL (*F* = 5.63; *p* = 0.025). Plasmatic concentrations of uric acid and total proteins were not affected (*F* = 1.30; *p* = 0.032) by animal group (nesting or non-nesting caiman). SVL did not affect uric acid concentration (*F* = 0.14; *p* = 0.719), but influenced total protein concentration (*F* = 5.86; *p* = 0.027).

### Regression analysis

Multiple regression analysis indicated that body condition did not affect (*F* = 2.18; *p* = 0.083) the plasmatic concentrations of glucose (*F* = 0.52; *p* = 0.472), lactate (*F* = 1.20; *p* = 0.280), uric acid (*F* = 0.60; *p* = 0.444) or total proteins (*F* = 2.71; *p* = 0.110) in nesting female *C. crocodilus*. However, there was a significant positive relationship (*F* = 6.93; *p* = 0.013) between body condition and plasmatic concentration of triglycerides. Linear regression indicated that body condition explained about 13% of variability in plasma triglyceride concentration in nesting female of *C. crocodilus* (*r* ^2^ = 0.13; 5.07; *p* = 0.03). In non-nesting *C. crocodilus*, multiple regression analysis indicated no relationship between body condition and the five blood plasma parameters evaluated (*F* = 0.46; *p* = 0.802). In *M. niger*, body condition had no significant effect on plasmatic concentrations of glucose and triglycerides in either nesting (*F* = 1.09; *p* = 0.368) or non-nesting caimans (*F* = 1.61; *p* = 0.244). Uric acid and total protein concentrations were also not significantly affected by body condition in nesting (*n* = 7; *F* = 0.32; *p* = 0.741) or non-nesting *M. niger* (*n* = 5; *F* = 1.63; *p* = 0.291).

Body condition of *C. crocodilus* was negatively affected (*r* ^2^ = 0.33; *F* = 5.58; *p* = 0.003) by nest distance to forest edge and nest age, and the relationships were described by the following equation: body condition = 2.734 − 0.010 × distance – 0.013 × age + 0.0002 × (distance × age). No significant effect (*F* = 1.86; *p* = 0.200) of nest distance to forest edge on female body condition was found for *M. niger*.

Effect of nest distance to forest edge was not significant for four of five serum biochemical values (*F* = 0.99; *p* = 0.441), and no significant effect of nest age was observed for any of the blood plasma parameters (*F* = 1.67; *p* = 0.176) in nesting female *C. crocodilus*. Nest distance explained about 18% of the variance in plasmatic glucose concentration in nesting female *C. crocodilus* (*r* ^2^ = 0.18; *F* = 7.67; *p* = 0.008). Low probability values in the linear regression of nest age on plasmatic concentrations of triglycerides, uric acid and total proteins in nesting female *C. crocodilus* indicated possible relationships for triglycerides (*r* ^2^ = 0.08; *F* = 2.98; *p* = 0.09), uric acid (*r* ^2^ = 0.08; *F* = 2.89; *p* = 0.09) and total proteins (*r* ^2^ = 0.09; *F* = 3.51; *p* = 0.07). In *M. niger*, no significant relationship (*F* = 0.52; *p* = 0.608) was found between nest distance to forest edge and plasmatic concentrations of glucose (*F* = 0.33; *p* = 0.574) or triglycerides (*F* = 0.67; *p* = 0.428). No effect of nest distance from forest edge on concentrations of uric acid and total proteins (*F* = 0.62; *p* = 0.596) was also observed in nesting female *M. niger*.

## Discussion

This study provides evidence that nest attendance has a metabolic cost for nesting females in *Caiman crocodilus*, but not in *Melanosuchus niger*. Body condition analysis and biochemical data indicated differences between the two species of caiman, which exhibit distinct nesting strategies even though they reproduce in the same region in the same season.

Body condition of an individual is directly influenced by its capacity to acquire resources required to support the metabolic costs (Jakob et al. 1996). The presence of factors, such as competition, food shortage or parental care, which may have a negative influence on individual aptitude to acquire resources, often results in a significant reduction of energetic reserves (Mrosovksy and Sherry 1980). In PP-SDR, nest attendance had a major influence on the ability of *C. crocodilus* to acquire resources, because body condition of 80% of females attending nests is lower than the mean body condition observed in females not attending nests. The lowest body condition value observed in non-nesting females was higher than those of 50% of nesting females.

For *M. niger* in MSDR, there was no apparent difference in body condition between nesting and non-nesting caimans, suggesting that nesting female *M. niger* do not lose body condition during nest attendance. Furthermore, and similar to what has been reported in other crocodilian species (Saafeld et al. 2008; Cedeño-Vasquez et al. 2011), there was no difference in body condition between males and non-nesting females in either locality. This similarity in body condition between males and females could be due to the fact that both sexes have access to the same resources, since males and non-nesting females usually inhabit in the same locations during the nesting season (Ayarzagüena 1983; Thorbjarnarson 1994). Several authors have suggested that variation in body condition in individuals of the same species between distinct geographical regions is influenced by habitat and food availability (Rootes et al. 1991; Saafeld et al. 2008), or conspecific density (Da Silveira et al. 1997).

The lower levels of plasmatic glucose, triglycerides and lactate in nesting female *C. crocodilus* than those of non-nesting individuals of the same species indicate that nest attendance during egg incubation has a direct impact on the energy reserves of the females, which affects their physiological status. Plasmatic triglyceride levels was the blood parameter that most differed between nesting and non-nesting *C. crocodilus*. Nearly 80% of non-nesting females and 70% of adult males exhibited higher triglyceride blood concentrations than the maximum value observed in nesting females.

Lower blood plasma concentrations of triglycerides in nesting female *C. crocodilus* suggest a metabolic cost associated with reproduction, which seems directly influenced by the species’ nesting strategy. Females remain near their nests in forests, during incubation period (Marioni et al. 2007; Ayarzagϋena and Castroviejo 2008), which ranges from 60 to 70 days (estimated for *Caiman crocodilus yacare* in the Pantanal; Cintra 1988) and alter their dietary composition and feed less often than non-nesting females (Barão-Nóbrega et al. 2016). Even though *M. niger* nesting females also remain near their nests during the incubation period, which ranges from 80 to 90 days (Villamarín et al. 2011), they are rarely seen on land, and usually remain in water near their nests (Thorbjarnarson et al. 2000), where they can remain hydrated and probably feed. Low concentrations of plasma triglycerides (between 0.1 and 0.2 mmol/L) have been documented for captive broad-snouted caiman (*Caiman latirostris*) that were fed with a low-fat diet (Zayas et al. 2011). There is also an association between low concentrations of plasmatic triglycerides and prolonged fasting periods in *Alligator mississippiensis* (Black et al. 1963) and *C. yacare* (Campbell et al. 2008).

None of the nesting female *C. crocodilus* exhibited plasmatic glucose concentrations higher than the mean value observed for non-nesting females and adult males (5.6 mmol/L) of the same species, whereas nesting and non-nesting *M. niger* exhibited similar values. Furthermore, about 85% of nesting females of *C. crocodilus* had a lower glucose concentration than the range (3.3 to 5.6 mmol/L) registered for the great majority of reptiles (Sykes and Klaphake 2008). In contrast, only 13% of nesting female *M. niger* exhibited a lower glucose value than 3.3 mmol/L. Nevertheless, it should be noted that comparison and interpretation of glucose concentrations might be limited when the fasting period is not known (Zayas et al. 2011). Dietary analysis of the stomach contents of the same group of nesting females of *C. crocodilus* demonstrated that their feeding frequency was significantly lower than non-nesting females (Barão-Nóbrega et al. 2016). Therefore, higher glucose concentrations in non-nesting individuals of *C. crocodilus* and both nesting and non-nesting individuals of *M. niger* could result from shorter fasting periods, since higher glucose values tend to occur when food has been ingested more recently (Lane 1996; Zayas et al. 2011).

As crocodilians exhibit low metabolism (Coulson and Herbert 1981) and it has been demonstrated in captivity that metabolic rates differ between animals recently fed and animals submitted to prolonged fasting periods (Gatten 1980), struggle associated with capture in non-nesting individuals of *C. crocodilus*, reflected in blood levels of lactate (Franklin et al. 2003; Hartzler et al. 2006), might have contributed to an increase in glucose concentrations (Coulson and Hernandez 1979; Lance et al. 1993). However, as our results demonstrated that longer capture efforts (i.e. more stressful to the animal) did not influence the biochemical parameters analysed, lower plasmatic lactate concentrations in nesting females of *C. crocodilus* indicate that nest attendance has another metabolic cost, making them less aggressive to stress factors, such as capture.

Although physiological consequences of dehydration in crocodilian species are still poorly understood (Ross 1989; Coppo et al. 2006), their effects are likely to be considerable. Nesting female *C. crocodilus* exhibited higher dehydration levels than adult males and non-nesting females of the same species. Ninety percent and 57% of the uric acid concentrations in nesting females were superior to the mean and maximum values observed for non-nesting individuals, respectively. Uric acid blood concentrations of nesting female *C. crocodilus* were also superior to those of nesting female *M. niger* in MSDR (where no difference between nesting and non-nesting individuals was observed) and to reference values recorded for other crocodilian species (Huchzermeyer 2003). Evidence of dehydration associated with nest attendance has also been reported in birds (Ankney and Afton 1988).

The positive association between body condition and plasma concentration of triglycerides in nesting female *C. crocodilus* indicates that they are using lipids as their primary source of metabolic energy (Campbell et al. 2008), and so depend on their accumulated lipid reserves to maintain their metabolic requirements during nest attendance, as has been suggested for the marine turtles *Chelonia mydas* and *Dermochelys coriacea* (Paladino et al. 1996; Hamann et al. 2002). During periods of low food intake, the main source of metabolic energy in *A. mississippiensis* (Black et al. 1963) and *C. yacare* (Campbell et al. 2008) originates from degradation of lipids rather than carbohydrates, such as glucose. On the other hand, in *M. niger*, the absence of correlation between triglycerides and body condition suggests that females of this species do not depend only on their lipid reserves while attending their nests during the incubation period, which could be directly related to their easier access to food resources (Campbell et al. 2008).

Differences observed in body condition and blood parameters between nesting and non-nesting individuals in *C. crocodilus* are likely to result from differences in feeding frequency and dietary composition (Campbell et al. 2008; Barão-Nóbrega et al. 2016), variation in prey availability (Taylor 1979; Delany and Abercrombie 1986) or foraging behaviour (Platt and Brantley 1991). In *A. mississippiensis*, a diet dominated by fish is correlated with a higher body condition (Delany et al. 1999) and nutritional status is directly related to biomass consumed (Chabreck 1971). Feeding frequency and occurrence of fish in the diet of nesting female *C. crocodilus* is very low (Barão-Nóbrega et al. 2016). Even though there is no available information regarding the diet of nesting female *M. niger*, it has been suggested that fish availability in lakes, where *M. niger* nesting females remain while attending their nests, could be higher than in the canals, due to lower water depth and currents (Da Silveira et al. 1997; Da Silveira and Magnusson 1999). Therefore, the absence of differences in body condition and serum biochemical values between nesting and non-nesting individuals of *M. niger* could be related to eating frequency and diet composition.

Although the reproductive biology and nesting ecology of *C. crocodilus* (Staton and Dixon 1977; Gorzula 1978; Thorbjarnarson 1994; Campos et al. 2008), and to a lesser extent *M. niger* (Thorbjarnarson et al. 2000; Villamarín and Suaréz 2007; Villamarín et al. 2011) have been studied, general knowledge on what influences body condition and internal physiology of females during the nesting season is still incipient. Our study demonstrates that nest location in the forest and duration of nest attendance both have negative impacts on female body condition in *C. crocodilus*. Female *C. crocodilus* attending nests in later stages of the incubation period or further into the forest have lower body condition than those attending nests closer to water or at earlier stages of the incubation period. Loss of body mass, and consequently body condition, during the incubation period has been reported also for nest-attending female birds (Mrosovsky and Sherry 1980; Ankney and Afton 1988; Kitaysky et al. 1999) and lizards (Huang 2007).

Similar to what has been observed in bird species (Owens and Bennet 1994; Carrillo and Aparicio 2001), nesting female *C. crocodilus* are exposed to greater depredation risk during nest attendance. In some regions of Central Amazonia, nesting female *C. crocodilus* and their eggs represent an important food source for jaguar, *Panthera onca* (Da Silveira et al. 2010). Despite the apparent negative impact on female body condition, locating their nests further inside the forest might be a strategy of *C. crocodilus* to decrease nest and attending-female detection by predators. Behavioural decisions with a cost/benefit associated with hatching success have been reported in *A. sinensis* in captivity (Zhang et al. 2015), birds (Cresswell 1997) and pythons (DeNardo et al. 2012). In MSDR, high predation rates were observed on eggs in nests of *C. crocodilus* and *M. niger* located close (up to 20 m) to the forest edge (72% of nests attacked in *C. crocodilus* and 50% in *M. niger*; Da Silveira et al. 2010). Freshwater crocodile (*Crocodylus johnstoni*) hole nests are more easily detected by predators when located close to the forest edge (Somaweera et al. 2011). In PP-SDR and Piranha-SDR (same type of habitat as our study sites, and located 175 km northeast of PP-SDR), where 80% of C. *crocodilus* nests are built inside the forest, at distances greater than 20 m from forest edge, the rate of attacks by predators on *C. crocodilus* nests seems to be lower (about 20%) than in MSDR (Barão-Nóbrega et al. 2014). However, comparing predation rates on eggs in crocodilian nests is not simple, because predation patterns may vary with predator and nest density, availability of alternative food resources for the predator and with the amount of maternal care given (Staton and Dixon 1977).

Besides the negative effect on body condition, both nest distance and age significantly influenced blood parameters of nesting female *C. crocodilus*, but not in *M. niger*. As blood glucose regulates the consumption rate of an organism (Kaneko 2008), the positive correlation between glucose and nest distance in *C. crocodilus* suggests that building nests further away from water demands higher energy expenditure by females. Time in attendance at nests likely has negative effects on plasmatic triglycerides, uric acid and total proteins concentrations. Even though these relations were not statistical significant at *p* < 0.05, the probability values were low, and we consider that they are likely to be biologically significant. Considerable reduction in lipid reserves during incubation is expected because lipids represent the major source of metabolic energy in crocodilians (Black et al. 1963), a pattern also described in birds that exhibit intensive parental care (Ankney and Afton 1988; Robin et al. 1988). Male and female imperator penguins (*Aptenodytes forsteri*) consume nearly 80% of their lipid reserves, which represents a loss of 20–40% in body mass, during the 3-month fasting period associated with reproduction (Robin et al. 1988).

As uric acid is one of the main by-products of protein catabolism in reptiles (Frye 1991), lower body condition associated with higher concentrations of uric acid in nesting female *C. crocodilus* suggests dehydration and/or some utilization of proteins as an additional energy source, especially because there seems to be a negative relationship (though not statistically significant) between nest age and total protein concentration (i.e. nesting female *C. crocodilus* seem to exhibit lower total protein concentrations in later stages of the incubation period). Nesting female *Anas clypeata* (Northern Shoveler) loose 15% of their body mass during the nesting season due to utilization of proteins as an additional energetic source (Ankney and Afton 1988).

Although *C. crocodilus* and *M. niger* share the same nesting seasons and build their nests in the same regions in Central Amazonian floodplains, they exhibit distinct nesting strategies. The nesting strategy of *C. crocodilus* seems to impose a metabolic cost associated with nest attendance because non-nesting individuals exhibited higher body condition and healthier serum biochemical values than those of nesting females. While nesting female *C. crocodilus* tend to remain hidden in the nearby vegetation, some in a lethargic state (Marioni et al. 2007), nesting female *M. niger* usually remain in water and aggressively defend their nests (Thorbjarnarson et al. 2000). Differences in parental care provided by nesting females of these species could be related to resource availability, as has been documented for nesting birds (Martin 1992; Dearborn 2001). Furthermore, nest attendance may be influenced by body condition (Dearborn 2001; Huang 2007) and the female’s accumulated energy reserves, since it has been suggested that nest defence behaviour and the period of nest attendance (i.e. how much time the female remains near the nest) may vary among nesting females in crocodilians (Kushlan and Kushlan 1980; Ayarzagüena 1983; Zhang et al. 2015).

Other aspects not addressed in this study such as female body condition prior to nesting (Brandt et al. 2016) and reproductive allometry also need to be taken into account. Associations between female body size and clutch characteristics have been reported in several crocodilians (Thorbjarnarson 1996), including *C. crocodilus* and *C. yacare* (Campos et al. 2008). In *C. latirostris*, it has been reported that energetic investment on reproduction may be higher in smaller females, than in larger females (Verdade 2001). Additionally, even though we have not observed evidence of nest-site selection (nest distance to standing water) within the forest in relation to female size (i.e. social hierarchy) in PP-SDR, it has been reported that it might influence nest-site selection in different habitats in *C. latirostris* (Montini et al. 2006). While there are still many questions to be answered, the values of indices of body condition and estimates of blood parameters for *C. crocodilus* and *M. niger* during the nesting season we registered provide reference values (Table 1) for future studies.
