# Correction to: The metabolic cost of nesting: body condition and blood parameters of *Caiman crocodilus* and *Melanosuchus niger* in Central Amazonia

**DOI:** 10.1007/s00360-017-1133-2

**Published:** 2017-11-13

**Authors:** José António Lemos Barão-Nóbrega, Boris Marioni, Robinson Botero-Arias, António José Arsénia Nogueira, Emerson Silva Lima, William Ernest Magnusson, Ronis Da Silveira, Jaydione Luiz Marcon

**Affiliations:** 1 Departamento de Biologia, Universidade de Aveiro - UA, Campus de Santiago, 3810-193 Aveiro, Portugal; 2 Programa de Pós-Graduação em Ecologia – Instituto Nacional de Pesquisas da Amazónia (INPA), CP 478, Manaus, AM 69011-970 Brazil; 3 Programa de Conservação de Crocodilianos Amazónicos – Instituto Piagaçu, Rua UZ no. 8 Cj Morada do Sol, Manaus, AM 6900-000 Brazil; 4 Programa de Pesquisa em Conservação e Manejo de Jacarés – Instituto de Desenvolvimento Sustentável Mamirauá, Estrada do Bexiga, 2584 - Bairro Fonte Boa, Tefé, AM CEP 69470-000 Brazil; 5 Department of Wildlife Ecology and Conservation, Institute of Food and Agricultural Sciences, University of Florida, 110 Newins-Ziegler Hall, PO Box 110430, Gainesville, FL 32611-0430 USA; 6 Centro de Estudos do Ambiente e do Mar - CESAM - UA, Campus de Santiago, 3810-193 Aveiro, Portugal; 7 Laboratório de Atividade Biológica, Faculdade de Ciências Farmacêuticas, Universidade Federal do Amazonas, Av. Gen, Rodrigo Otávio 6200, Manaus, AM CEP 69077-070 Brazil; 8 Coordenação de Biodiversidade, Instituto Nacional de Pesquisas da Amazónia (INPA), CP 2228, Manaus, AM 69080-971 Brazil; 9 Laboratório de Zoologia-Aplicada à Conservação, Departamento de Biologia, Instituto de Ciências Biológicas, Universidade Federal do Amazonas, Av. Gen, Rodrigo Otávio 6200, Manaus, AM CEP 69077-070 Brazil; 10 Laboratório de Fisiologia Animal, Departamento de Ciências Fisiológicas, Instituto de Ciências Biológicas, Universidade Federal do Amazonas, Av. Gen, Rodrigo Otávio 6200, Manaus, AM CEP 69077-070 Brazil

## Correction to: J Comp Physiol B 10.1007/s00360-017-1103-8

The article “The metabolic cost of nesting: body condition and blood parameters of Caiman crocodilus and Melanosuchus niger in Central Amazonia”, written by José António Lemos Barao‑Nobrega, Boris Marioni, Robinson Botero‑Arias, António José Arsénia Nogueira, Emerson Silva Lima, William Ernest Magnusson, Ronis Da Silveira, Jaydione Luiz Marcon was originally published electronically on the publisher’s internet portal (currently SpringerLink) on June 19, 2017 without open access.

With the author(s)’ decision to opt for Open Choice the copyright of the article changed on November 1, 2017_The Author(s) [2017] and the article is forthwith distributed under the terms of the Creative Commons Attribution [continuing for CC BY license].

4.0 International License (http://creativecommons.org/licenses/by/4.0/), which permits use, duplication, adaptation, distribution and reproduction in any medium or format, as long as you give appropriate credit to the original author(s) and the source, provide a link to the Creative Commons license, and indicate if changes were made.

The original article has been corrected.

